# Induction of glucose uptake in skeletal muscle by central leptin is mediated by muscle β_2_-adrenergic receptor but not by AMPK

**DOI:** 10.1038/s41598-017-15548-6

**Published:** 2017-11-09

**Authors:** Tetsuya Shiuchi, Chitoku Toda, Shiki Okamoto, Eulalia A. Coutinho, Kumiko Saito, Shinji Miura, Osamu Ezaki, Yasuhiko Minokoshi

**Affiliations:** 1 Division of Endocrinology and Metabolism, Department of Homeostatic Regulation, National Institute for Physiological Sciences, National Institutes of Natural Sciences, Okazaki, Aichi 444-8585 Japan; 2 Department of Physiological Sciences, School of Life Sciences, SOKENDAI (The Graduate University for Advanced Studies), Okazaki, Aichi 444-8585 Japan; 3 Department of Integrative Physiology, Institute of Biomedical Sciences, Tokushima University Graduate School, Tokushima, 770-8503 Japan; 4 Nutritional Science Program, National Institute of Health and Nutrition, Tokyo, 162-8636 Japan; 5 Present Address: Laboratory of Biochemistry, Graduate School of Veterinary Medicine, Hokkaido University, Sapporo, 060-0818 Japan; 6 Present Address: Second Department of Internal Medicine (Endocrinology, Diabetes and Metabolism, Hematology, Rheumatology), Graduate School of Medicine, University of the Ryukyus, Okinawa, 903-0215 Japan; 7 Present Address: Centre for Neuroendocrinology and Department of Physiology, School of Biomedical Sciences, University of Otago, Dunedin, 9054 New Zealand; 8 Present Address: Laboratory of Nutritional Biochemistry, Graduate School of Nutritional and Environmental Sciences, University of Shizuoka, Shizuoka, 422-8526 Japan; 9 Present Address: Department of Human Health and Design, Showa Women’s University, Tokyo, 154-8533 Japan

## Abstract

Leptin increases glucose uptake and fatty acid oxidation (FAO) in red-type skeletal muscle. However, the mechanism remains unknown. We have investigated the role of β_2_-adrenergic receptor (AR), the major β-AR isoform in skeletal muscle, and AMPK in leptin-induced muscle glucose uptake of mice. Leptin injection into the ventromedial hypothalamus (VMH) increased 2-deoxy-D-glucose (2DG) uptake in red-type skeletal muscle in wild-type (WT) mice accompanied with increased phosphorylation of the insulin receptor (IR) and Akt as well as of norepinephrine (NE) turnover in the muscle. Leptin-induced 2DG uptake was not observed in β-AR-deficient (β-less) mice despite that AMPK phosphorylation was increased in the muscle. Forced expression of β_2_-AR in the unilateral hind limb of β-less mice restored leptin-induced glucose uptake and enhancement of insulin signalling in red-type skeletal muscle. Leptin increased 2DG uptake and enhanced insulin signalling in red-type skeletal muscle of mice expressing a dominant negative form of AMPK (DN-AMPK) in skeletal muscle. Thus, leptin increases glucose uptake and enhances insulin signalling in red-type skeletal muscle via activation of sympathetic nerves and β_2_-AR in muscle and in a manner independent of muscle AMPK.

## Introduction

Leptin inhibits food intake and increases energy expenditure in animals^1,2^. It also increases glucose utilization and fatty acid oxidation (FAO) in certain peripheral tissues including skeletal muscle, without affecting plasma glucose and insulin levels, in rodents^3–8^. In addition, leptin markedly ameliorates metabolic abnormalities associated with type 2 diabetes mellitus in humans and animals with lipodystrophy by increasing insulin sensitivity in peripheral tissues^9,10^, and it alleviates streptozotocin-induced type 1 diabetes in rodents^11^. The mechanism by which leptin increases glucose uptake in peripheral tissues has remained incompletely understood, however.

Skeletal muscle is a key tissue in whole-body energy metabolism and is responsible for insulin resistance associated with obesity and type 2 diabetes^12^. Glucose and lipid metabolism in skeletal muscle are regulated by insulin, but also by the central nervous system. We and other thus previously showed that peripheral injection of leptin or leptin injection into the ventromedial hypothalamus (VMH) of rodents increases glucose uptake and insulin sensitivity in red-type skeletal muscle such as the soleus as well as in heart muscle and BAT (brown adipose tissue), but not in white adipose tissue (WAT), without change in plasma glucose and insulin level^3–5,7^. These effects of peripheral leptin were abolished by injection of an inhibitor of extracellular signal–regulated kinase (ERK) signalling into the VMH^8^. Specific ablation of the leptin receptor in steroidogenic factor 1 (SF1)-positive cells in the VMH induced obesity and increased susceptibility to a high-fat diet in mice^13^. Recent study also showed that SF1 expression in the VMH is required for beneficial metabolic effects of exercise^14^. Furthermore, we recently showed that activation of SF1 neurons in the VMH by DREADD (Designer Receptors Exclusively Activated by Designer Drug) technology increases insulin sensitivity in red-type of skeletal muscle, heart and BAT, but not WAT^15^.

To examine the effect of leptin injection and activation of SF1 neurons by DREADD on insulin sensitivity in the peripheral tissues, we performed hyperinsulinemic-euglycemic clamp^8,15^. In basal period, activation of SF1 neurons by DREADD as well as peripheral or VMH injection of leptin increased Rd (glucose disappearance rate) and glucose uptake in red-type skeletal muscles, heart and BAT. This effect was accompanied by an increase in Ra (glucose appearance rate) and activation of hepatic phosphorylase a activity, thereby maintaining blood glucose level. In hyperinsulinemic-euglycemic clamp period, leptin strongly increased Rd and glucose uptake in the same tissues, and Ra was suppressed by inhibiting phosphorylase a activity and mRNA expression of PEPCK and G6Pase. Thus, our results suggested that VMH leptin increases whole-body glucose turnover during basal period and insulin sensitivity in some peripheral tissues including red-type skeletal muscle.

Activation of sympathetic nerves and β-adrenergic receptors (β-ARs) is required for leptin-induced glucose uptake in peripheral tissues^5^. The β-AR antagonist propranolol and guanethidine, a blocker of sympathetic nerve activity, were thus each shown to attenuate this effect of leptin^5^. Activation of sympathetic nerves and β_2_-AR are also necessary for the exercise-induced increase in peroxisome proliferator–activated receptor–γ coactivator–1α (PGC-1α) mRNA abundance in skeletal muscle^16^. However, whether leptin-induced glucose uptake in skeletal muscle also requires muscle β_2_-ARs has remained unknown. Previous studies revealed that β-AR agonist increases glucose uptake in skeletal muscle^17,18^, but others showed that catecholamines inhibits or has no effect on glucose uptake in skeletal muscle^19,20^.

AMP-activated protein kinase (AMPK) functions as a cellular “fuel gauge” and its activation stimulates glucose uptake and FAO in skeletal muscle^21^. We previously showed that injection of leptin into the medial hypothalamus including the arcuate nucleus of the hypothalamus (ARH) and VMH increased FAO in red-type skeletal muscle via activation of AMPK as well as of sympathetic nerves and α-ARs in the muscle tissue^6^. AMPK activation is sufficient to mimic exercise- or muscle contraction–induced glucose uptake in skeletal muscle^22–25^.

We have now examined the roles of β_2_-AR, the major β-AR isoform in skeletal muscle^26,27^, and of AMPK in leptin-induced glucose uptake and enhancement of insulin signalling in skeletal muscle. The central effects of leptin were evaluated in β-AR–deficient (β-less) mice^28^ and in mice expressing a dominant negative form of AMPK (DN-AMPK) specifically in skeletal muscle^29^. The β_2_-AR was forcibly expressed in red-type skeletal muscle in the right hind limb of β-less mice. Our results suggest that leptin injection into the VMH increases glucose uptake and enhances insulin signalling in red-type skeletal muscle via β_2_-AR, but not via AMPK. Thus, our findings provide an important insight into regulation of glucose metabolism by the central nervous system and its potential for therapeutic manipulation.

## Results

### Leptin increases glucose uptake in peripheral tissues of WT but not β-less mice

**Figure 1:**
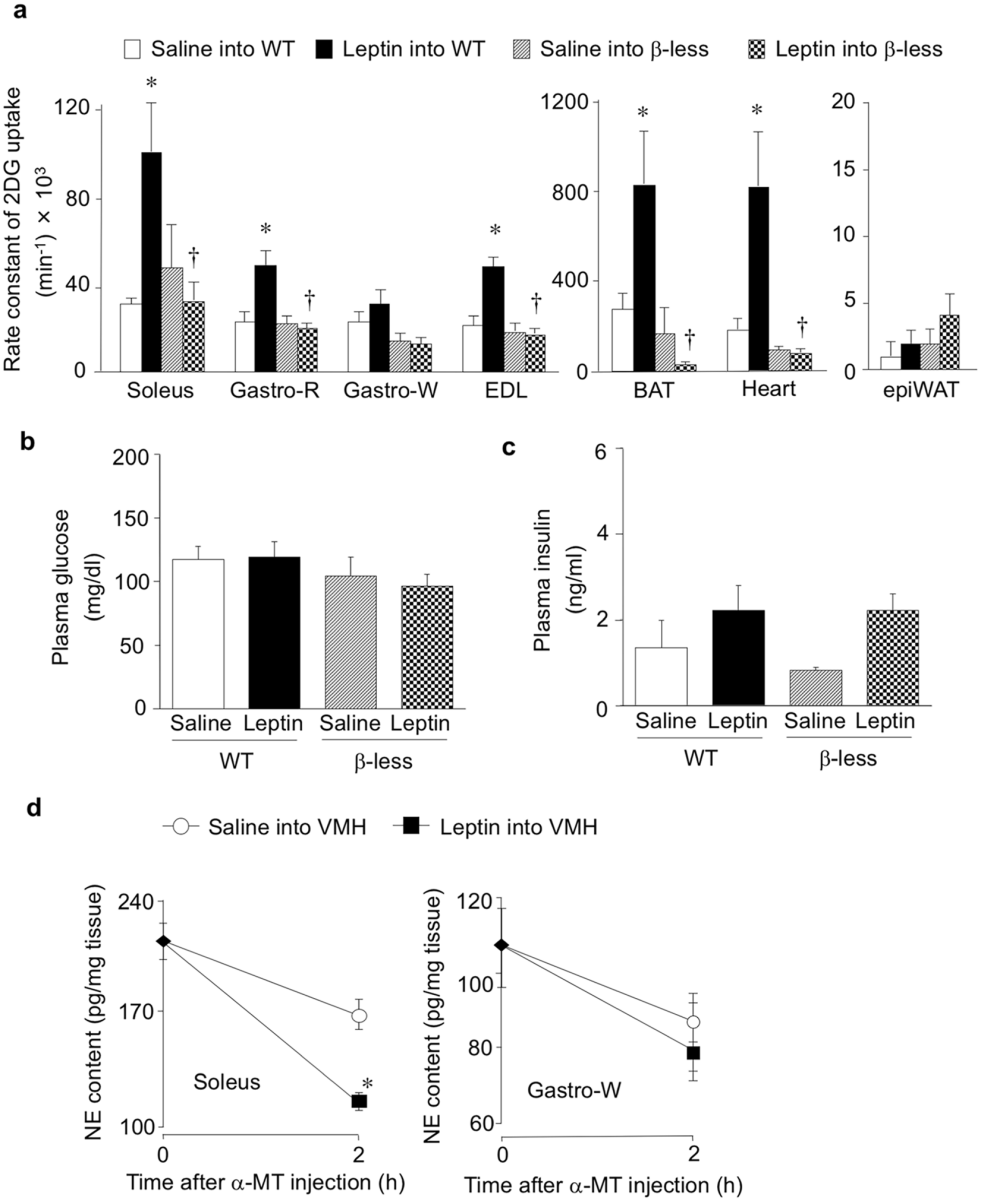
Leptin injection into the VMH increases 2DG uptake in certain peripheral tissues of WT mice but not in those of β-less mice. (**a**) Rate constant of 2DG uptake in peripheral tissues of WT and β-less mice measured 6 h after injection of saline or leptin into the VMH (*n* = 6). Gastro-R: red portion of gastrocnemius, Gastro-W: white portion of gastrocnemius, EDL: extensor digitorum longus, BAT: brown adipose tissue, epiWAT: epididymal white adipose tissue. (**b**,**c**) Plasma glucose (**b**) and insulin (**c**) concentrations in WT and β-less mice at 6 h after injection of leptin into the VMH (*n* = 6). (**d**) NE turnover in soleus and Gastro-W muscles of WT mice measured 6 h after saline or leptin injection into the VMH (*n* = 6 or 7) (unpaired Student’s *t* test). α-MT: α-methyl-*p*-tyrosine. All data are means ± S.E.M. *P < 0.05 versus corresponding value for saline injection into WT mice; ^†^ *P* < 0.05 versus corresponding value for leptin injection into WT mice (one-way ANOVA and Bonferroni’s multiple-range test).

We examined the effects of leptin injection into the VMH on glucose uptake in peripheral tissues of β-less and WT mice. Consistent with previous observations^4,5,7,30^, the rate constant of 2DG uptake, a marker of glucose uptake activity, was significantly increased in red-type [soleus and Gastro-R (red portion of gastrocnemius)] and mixed-type (EDL, extensor digitorum longus) skeletal muscle, but not in Gastro-W (white portion of gastrocnemius) or epiWAT (epididymal white adipose tissue) of WT mice at 6 h after leptin injection (Fig. 1a). Leptin also increased the rate constant of 2DG uptake in heart muscle and BAT but not in epididymal WAT of WT mice. In contrast, leptin did not increase 2DG uptake in any of these peripheral tissues of β-less mice (Fig. 1a). Consistent with the previous reports^3,4,7,8,31,32^, plasma glucose and insulin concentrations were not affected by leptin injection into the VMH of WT or β-less mice (Fig. 1b,c). Injection of leptin into the VMH significantly increased NE turnover in soleus muscle but not in Gastro-W of WT mice (Fig. 1d). Norepinephrine (NE) turnover was measured on the basis of the decline in tissue NE content after the inhibition of catecholamine biosynthesis with α-methyl-*p*-tyrosine (α-MT) to evaluate sympathetic nerve activity of individual tissue in the same animals^33^. The results suggest that leptin increases the activity of sympathetic nerves innervating red-type but not white-type skeletal muscle. Thus leptin injection into the VMH increases glucose uptake in red-type skeletal muscle preferentially through activation of sympathetic nerves and β-AR in the tissue.

### Effects of leptin on insulin signalling and AMPK activity in soleus of WT and β-less mice

**Figure 2:**
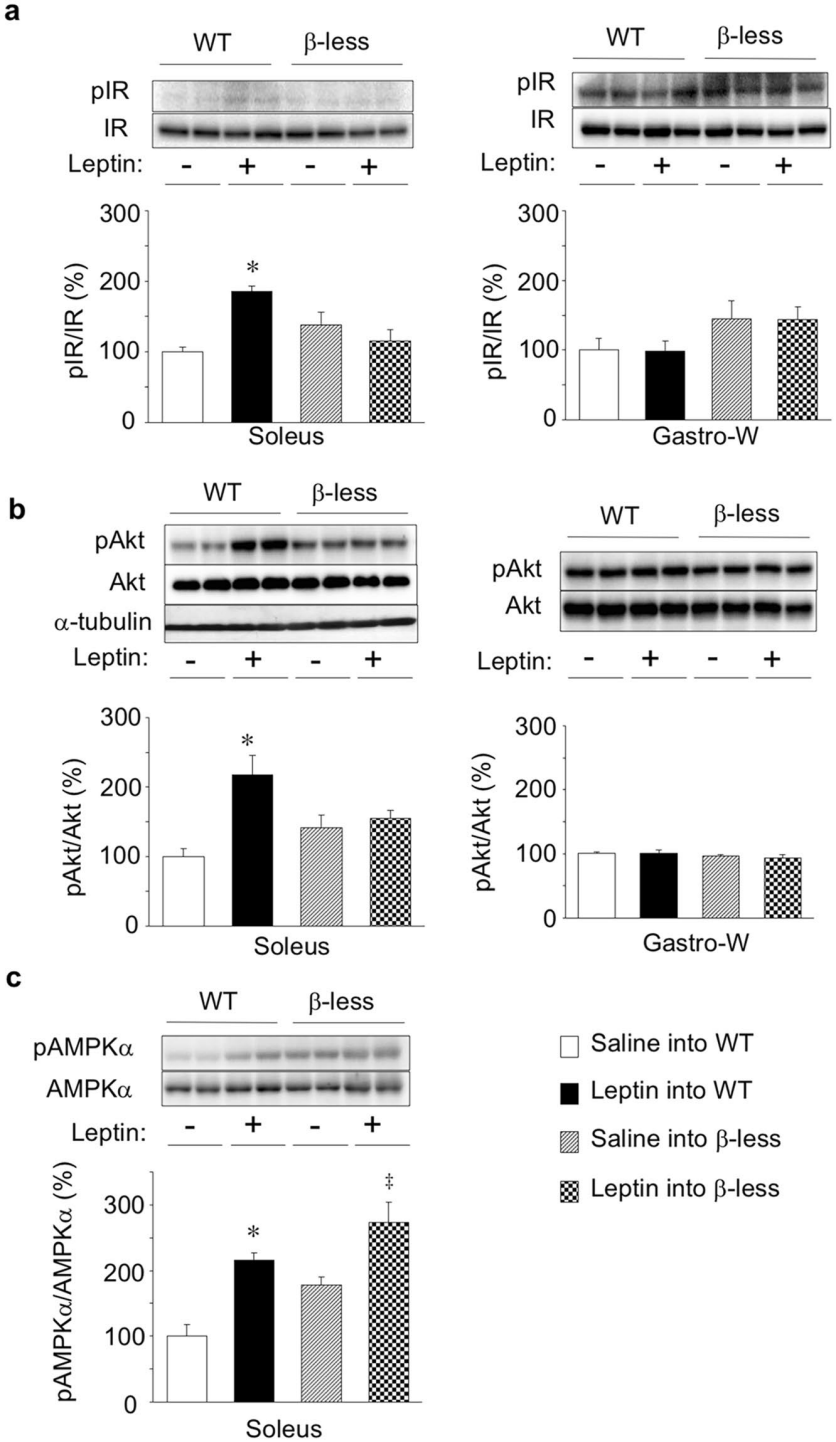
Effects of leptin on insulin signalling and AMPK activity in soleus muscle of WT and β-less mice. Representative immunoblot analysis of phosphorylated (p) and total forms of IR (**a**), Akt (**b**), and the α subunit of AMPK (**c**) in soleus or Gastro-W at 6 h after injection of saline (−) or leptin (+) into the VMH of WT or β-less mice is shown together with quantitation of the corresponding pIR/IR, pAkt/Akt, and pAMPKα/AMPKα ratios. Representative data were shown in duplicate. Representative immunoblots for α-tubulin were also shown in. (**b**) Quantitative data are expressed as a percentage of the corresponding value for injection of saline into WT mice and are means ± S.E.M. (*n* = 4). *P < 0.05 versus corresponding value for saline injection into WT mice; ^‡^ *P* < 0.05 versus corresponding value for saline injection into β-less mice (one-way ANOVA and Bonferroni’s multiple-range test).

To elucidate the mechanism by which leptin increases glucose uptake in red-type skeletal muscle, we examined the effect of leptin injection into the VMH on insulin signalling in skeletal muscle. Injection of leptin into the VMH increased the amounts of phosphorylated forms of IR and Akt in soleus muscle but not in Gastro-W of WT mice (Fig. 2a,b). In contrast, leptin did not increase the abundance of phosphorylated IR or Akt in soleus or Gastro-W of β-less mice (Fig. 2a,b). The extents of Akt as well as α-tubulin were not different among groups (Fig. 2b). Leptin thus enhanced insulin signalling in red-type skeletal muscle in a β-AR-dependent manner, similar to its effect on glucose uptake in such muscle.

AMPK is activated by phosphorylation at Thr^172^ of its α subunit by AMPK kinases such as LKB1 and Ca^2+^- and calmodulin-dependent protein kinase kinase (CaMKK)^34,35^. Leptin induces AMPK phosphorylation in red-type skeletal muscle including soleus^5^. We found that injection of leptin into the VMH increased the extent of AMPKα phosphorylation but not AMPKα in soleus muscle of both WT and β-less mice (Fig. 2c), suggesting that AMPK activation in red-type skeletal muscle is not sufficient for leptin-induced glucose uptake or enhancement of insulin signalling in this tissue as well as that leptin activates AMPK in skeletal muscle through a β-AR–independent mechanism.

### Leptin increases muscle glucose uptake via β_2_-AR in muscle

**Figure 3:**
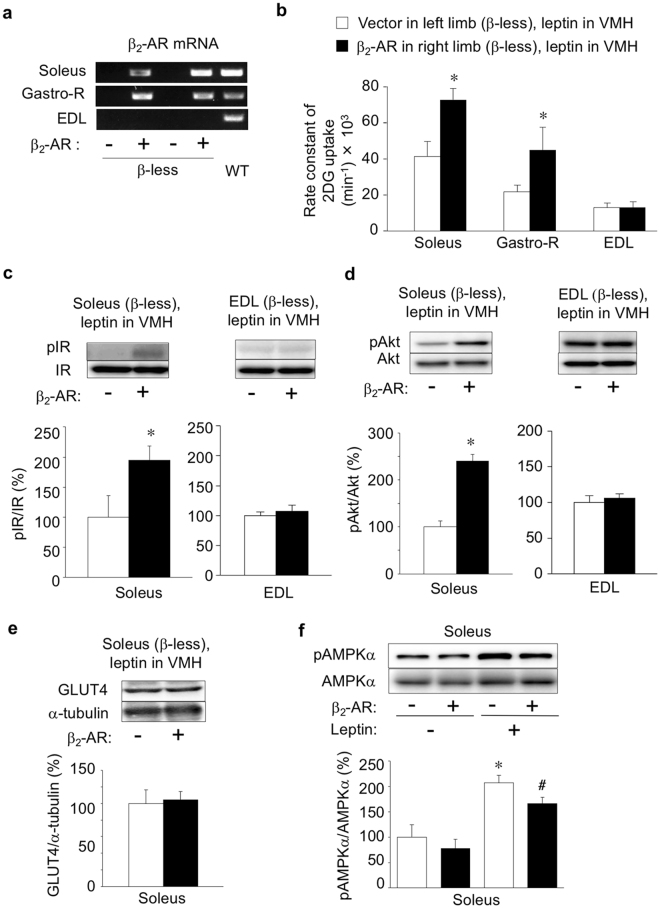
Forced expression of β_2_-AR in red-type skeletal muscle of β-less mice restores leptin-induced glucose uptake. (**a**) RT-PCR analysis of β_2_-AR mRNA in soleus, Gastro-R, and EDL muscles of β-less mice at 8 days after *in vivo* electroporation with an expression vector for β_2_-AR in the soleus and Gastro-R of the right hind limb (+) as well as with the corresponding empty vector in the same muscles of the left hind limb (−). Representative data for two manipulated β-less mice and one intact WT mouse are shown. (**b**) Rate constant of 2DG uptake in soleus, Gastro-R, and EDL muscles of the left (−) and right (+) hind limbs of β-less mice (*n* = 5) measured at 8 days after *in vivo* electroporation as in (**a**) and at 6 h after leptin injection into the VMH. (**c**,**d**) Representative immunoblot analysis of phosphorylated (p) and total forms of IR (**c**) and Akt (**d**) in soleus and EDL muscles of the (−) and (+) hind limbs of β-less mice treated as in (**b**) is shown together with quantitation of the corresponding pIR/IR and pAkt/Akt ratios (*n* = 4). (**e**) Representative immunoblot analysis and quantitation (*n* = 4) of GLUT4 and α-tubulin in soleus muscle of the (−) and (+) hind limbs of β-less mice treated as in (**b**). *P < 0.05 versus corresponding value for the β_2_-AR(−) muscle in the left hind limb (paired Student’s *t* test). (**f**) Representative immunoblot analysis and quantitation (*n* = 4) of phosphorylated (pAMPKα) and total (AMPKα1 and AMPKα2) forms of the α1 and α2 subunits of AMPK in soleus muscle of the (−) and (+) hind limbs of β-less mice measured at 8 days after *in vivo* electroporation as in (**a**) and at 6 h after leptin (+) or saline (−) injection into the VMH. *P < 0.05 versus corresponding value for the β_2_-AR(−) muscle after saline injection; ^#^ *P* < 0.05 versus corresponding value for the β_2_-AR(+) muscle after saline injection. All quantitative data are means ± S.E.M.

The β_2_-AR is the major β-AR isoform in skeletal muscle of rodents and humans^26,27^. To examine the role of β_2_-AR in the leptin-induced increase in glucose uptake and enhancement of insulin signalling in red-type skeletal muscle, we restored expression of β_2_-AR in soleus and Gastro-R muscles in the right hind limb of β-less mice by *in vivo* electroporation with an expression construct controlled by the CAG promoter. The abundance of β_2_-AR mRNA in the right soleus and Gastro-R muscles of β-less mice at 8 days after electroporation was similar to that in the corresponding muscles of WT mice (Fig. 3a). The amount of β_2_-AR mRNA was not similarly increased in EDL muscle of the right hind limb in the electroporated mice. Injection of leptin into the VMH increased the rate constant of 2DG uptake in the right soleus and Gastro-R, but not in the right EDL, of the electroporated β-less mice, compared with that apparent for the contralateral muscles not expressing β_2_-AR (Fig. 3b). Furthermore, central leptin injection increased the amounts of phosphorylated IR and Akt in the β_2_-AR–expressing right soleus but not in the β_2_-AR–deficient right EDL muscle (Fig. 3c,d). The amount of GLUT4 protein did not change in the β_2_-AR–expressing soleus muscle compared with that in β_2_-AR–deficient muscle (Fig. 3e). In contrast, leptin increased the level of AMPK phosphorylation in both the β_2_-AR–expressing right soleus and the β_2_-AR–deficient left soleus of β-less mice (Fig. 3f). These results thus suggested that leptin-induced glucose uptake and enhancement of insulin signalling in red-type skeletal muscle are mediated by muscle β_2_-AR, whereas leptin-induced AMPK activation is not.

### Leptin increases glucose uptake and enhances insulin signalling in skeletal muscle in an AMPK-independent manner

**Figure 4:**
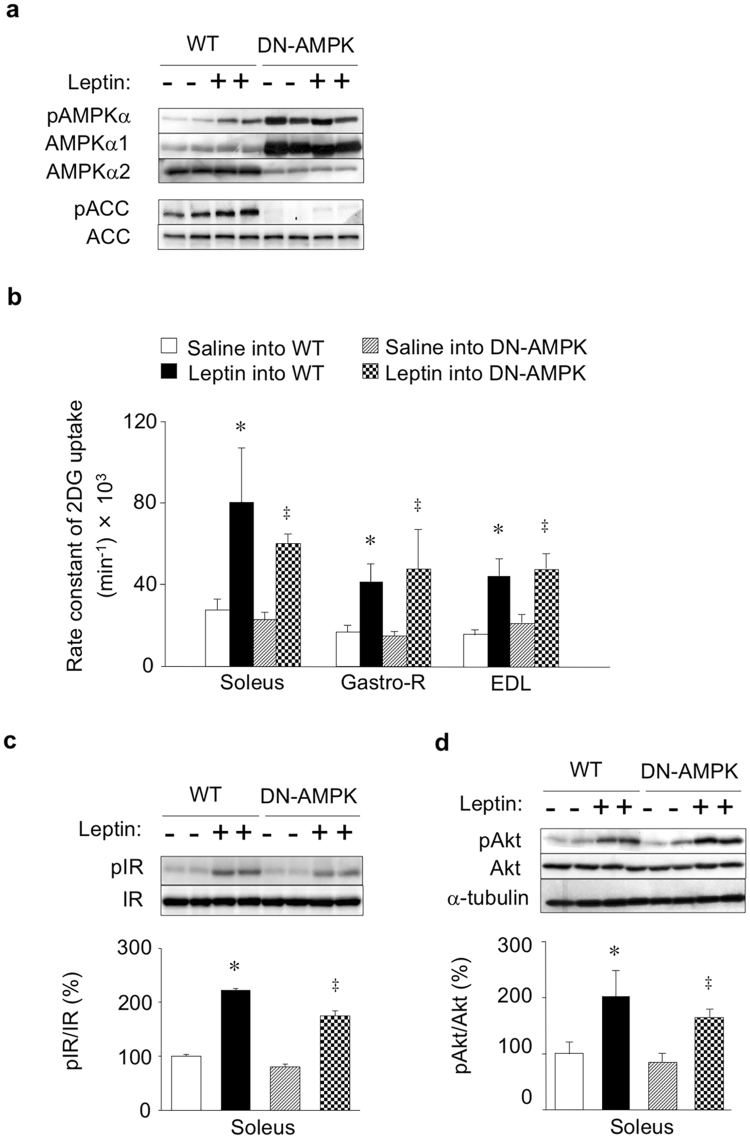
Leptin increases glucose uptake and enhances insulin signalling in skeletal muscle of DN-AMPK mice. (**a**) Representative immunoblot analysis of phosphorylated (pAMPKα) and total (AMPKα1 and AMPKα2) forms of the α1 and α2 subunits of AMPK as well as of phosphorylated and total ACC in soleus muscle of WT and DN-AMPK mice at 6 h after injection of saline (−) or leptin (+) into the VMH. (**b**) Rate constant of 2DG uptake in soleus, Gastro-R, and EDL muscles at 6 h after injection of saline or leptin into the VMH of WT or DN-AMPK mice (*n* = 5). (**c**, **d**) Representative immunoblot analysis and quantitation (*n* = 4) of pIR/IR (**c**) and pAkt/Akt (**d**) in soleus muscle of WT or DN-AMPK mice at 6 h after injection of saline (−) or leptin (+) into the VMH. Representative immunoblots for α-tubulin were also shown in. (**d**) All quantitative data are means ± S.E.M. *P < 0.05 versus corresponding value for saline injection in WT mice. ^‡^ *P* < 0.05 versus corresponding value for saline injection in DN-AMPK mice (one-way ANOVA and Bonferroni’s multiple-range test).

Finally, we examined the effect of suppression of AMPK activity in skeletal muscle on leptin-induced glucose uptake in red-type skeletal muscle with the use of DN-AMPK transgenic mice, which express a dominant negative form of the α1 subunit of AMPK in skeletal muscle^29^. Consistent with previous observations^29^, the soleus muscle of DN-AMPK mice manifested marked increases in both the amount of the α1 subunit of AMPK and the level of α subunit phosphorylation (at Thr^172^) as well as a decrease in the abundance of the α2 subunit (Fig. 4a), with the latter effect thought to be the result of degradation of the α2 subunit excluded from the heterotrimeric complex of AMPK^29^. Leptin injection into the VMH increased the level of Ser^79^-phosphorylation of the AMPK substrate ACC in soleus muscle of WT mice but not in that of DN-AMPK mice (Fig. 4a). Expression of DN-AMPK in skeletal muscle was previously shown to suppress signalling downstream of AMPK^29,36^. Nevertheless, leptin injection into the VMH increased the rate constant of 2DG uptake in soleus, Gastro-R, and EDL muscles of DN-AMPK mice to an extent similar to that apparent in WT mice (Fig. 4b). The leptin-induced increases in the extents of IR and Akt phosphorylation as well as total Akt protein in soleus muscle were also similar in DN-AMPK and WT mice (Fig. 4c,d). These results thus suggested that activation of AMPK in skeletal muscle is not necessary for leptin-induced glucose uptake and enhancement of insulin signalling in this tissue.

## Discussion

We have here shown that leptin injection into the VMH increases glucose uptake in red-type skeletal muscle through activation of sympathetic nerves and β_2_-AR in the muscle tissue. This effect of leptin was thus not apparent in β-less mice, whereas forced expression of β_2_-AR in red-type skeletal muscle of these mice restored both the leptin-induced increase in glucose uptake and enhancement of insulin signalling. We also found that leptin activates sympathetic nerves innervating red-type but not white-type skeletal muscle. These results suggest that leptin-induced glucose uptake in red-type skeletal muscle is mediated by the activation of sympathetic nerves and β_2_-AR in the muscle tissue.

We previously showed that injection of orexin into the VMH of mice activates VMH neurons and promotes insulin-induced glucose uptake in red-type skeletal muscle including soleus and Gastro-R via activation of sympathetic nerves and β_2_-AR in muscle tissue^30^. This effect of orexin was thus not apparent in β-less mice, whereas forced expression of β_2_-AR under the control of the CAG promoter in both red-type myocytes and nonmyocytes including blood vessel cells in these mice restored the effect. Activation of β_2_-AR in blood vessels stimulates vascular relaxation^37^ and thereby increases insulin delivery to myocytes^38^, which plays a key role in muscle glucose disposal^39^. In the present study, forced expression of β_2_-AR in red-type skeletal muscle of β-less mice restored phosphorylation of IR and Akt in the muscle tissue in response to leptin administration into the VMH. Thus such leptin injection may stimulate glucose uptake in red-type skeletal muscle via enhancement of insulin signalling in a manner dependent on β_2_-AR–induced vasodilation in the muscle vasculature and a consequent increase in insulin delivery to myocytes. Indeed, we previously showed that leptin-induced muscle glucose uptake is blunted by the nitric oxide synthase inhibitor L-NAME [N:(G)-nitro-L-arginine methyl ester]^40^. It is also possible, however, that humoral factors might contribute to the action of leptin through cooperative activation of insulin or other signalling pathways in skeletal muscle.

Leptin may partly increase glucose uptake in skeletal muscle by β_2_-AR expressed in myocytes and through an insulin-independent mechanism. We previously showed that forced expression of β_2_-AR under the control of the myocyte-specific promoter HSA (human α-skeletal actin) partly rescued the orexin-induced glucose uptake in soleus and Gastro-R muscles in β-less mice in an insulin-independent manner^30^. HSA promoter expressed β_2_-AR in the red-type muscles, similar to the level in muscle expressed β_2_-AR by CAG promoter. Orexin injection into the VMH increased glucose uptake in red-type skeletal muscles without change in phosphorylation of IR. However, the orexin-induced glucose uptake in muscles expressed β_2_-AR in myocytes was significantly smaller than that in muscles expressed β_2_-AR by CAG promoter^30^. Furthermore, the leptin-induced muscle glucose uptake was abolished by L-NAME^40^. Thus, although β_2_-AR in myocytes might be involved in the leptin-induced glucose uptake in red-type skeletal muscle, β_2_-AR in nonmyocytes, including blood vessels, plays an important role in the leptin-induced activation of insulin signalling including IR-Akt and glucose uptake in red-type of skeletal muscle *in vivo*. Further investigation is necessary to clarify the role of β_2_-AR in myocytes and blood vessels in leptin-induced glucose uptake in the muscle tissue.

It is possible that the β_1_-AR in red-type skeletal muscles might also regulate the leptin-induced glucose uptake in skeletal muscle of WT mice, despite its low expression red-type skeletal muscle tissue^30^ compared to β_1_-AR expression in heart muscle and BAT and β_2_-AR in skeletal muscle. Further investigation to examine the effect of β_1_-AR on glucose uptake in the muscle would be needed. Nevertheless, the present results showed that expression of β_2_-AR sufficiently rescues the leptin-induced glucose uptake in red-type skeletal muscle in β-less mice without expression of β_1_ and β_3_-AR in the skeletal muscle tissue.

Previous reports showed that AMPK activation is sufficient but not required for exercise- or muscle contraction–induced glucose uptake in skeletal muscle^17,18^. Consistent with the studies, AMPK was unlikely to be a primary mediator of leptin-induced glucose uptake in red-type skeletal muscle. However, β_2_-AR may regulate phosphorylation of AMPK in soleus muscle. pAMPK/AMPK ratio in soleus muscle of β-less mice tended to increase in the absence and presence of leptin injection into the VMH, compared with that in WT mice. Forced expression of β_2_-AR tended to decrease the leptin-induced phosphorylation of AMPK in the tissue. β_2_-AR thus appears to inhibit AMPK activity in soleus muscle. It is possible that change in AMPK activity in β-less mice modulates insulin signalling and glucose uptake in skeletal muscle in the mice. Further investigation is necessary to elucidate the role of AMPK in insulin signalling and glucose uptake in peripheral tissues in β-less mice.

Collectively, our results suggest that leptin requires sympathetic nerve activation and β_2_-AR stimulation in muscle tissue to increase glucose uptake in red-type skeletal muscle. In contrast, AMPK activation is not required for this effect of leptin. Leptin is effective for the treatment of type 2 diabetes in humans and animals with lipodystrophy^9,10^, and it ameliorates streptozotocin-induced type 1 diabetes in rodents^11^. Our findings provide a fuller understanding of the mechanism by which leptin stimulates glucose uptake in peripheral tissues and therefore offer new insight into regulation of glucose metabolism by the central nervous system and its potential for therapeutic manipulation.

## Materials and Methods

### Animals and surgery

Male β-less mice^28^ and skeletal muscle–specific DN-AMPK transgenic mice^29^, as well as their wild-type (WT) counterparts, were studied at 12 to 18 weeks of age. The DN-AMPK transgenic mice express a dominant negative form of rat AMPK α1 subunit with an Asp^157^-to-Ala mutation) in skeletal muscle under the control of the skeletal muscle–specific promoter of the human skeletal actin gene. All animals were housed individually in plastic cages at 24° ± 1 °C with lights on from 0600 to 1800 hours and with free access to a laboratory diet (MF; Oriental Yeast, Tokyo, Japan) and water. All animal experiments were approved by the Institutional Animal Care and Use Committee (IACUC) of the National Institutes of Natural Sciences (Okazaki, Japan), and they were performed according to institutional guidelines concerning the care and handling of experimental animals.

Mice were anesthetized by intraperitoneal injection of ketamine (100 mg/kg) and xylazine (10 mg/kg) for unilateral implantation of a chronic stainless steel cannula (Unique Medical, Osaka, Japan) into the VMH according to the stereotaxic coordinates AP 1.5 (1.5 mm anterior to the bregma), L 0.3 (0.3 mm lateral to the bregma), and H 5.8 (5.8 mm below the bregma) for the cannula tip. Leptin (50 pmol) (Peprotech, Rocky Hill, NJ) dissolved in 0.2 μl of physiological saline or saline alone as a control was injected via the cannula into the VMH of conscious, unrestrained mice with the use of a Hamilton microsyringe. A silicone catheter was implanted into the right atrium through the external jugular vein as described previously^30^. Animals were handled repeatedly during the recovery period (~2 weeks) after cannula implantation to habituate them to the injection and blood sampling procedures. All animal experiments were performed 6 h after saline or leptin injection into the VMH during the light period. Food, but not water, was removed immediately after injection of leptin or saline into the VMH.

### Measurement of glucose uptake

For measurement of glucose uptake in peripheral tissues, a mixture of 6.25 μCi of 2-deoxy-D-[^3^H]glucose (2DG) (10 Ci/mmol) and 1.25 μCi of [^14^C]sucrose (10 Ci/mmol) (American Radiolabeled Chemicals, St. Louis, MO) dissolved in 50 μl of saline was injected through the jugular vein catheter 6 h after microinjection of leptin or saline into the VMH. Blood was collected for isolation of plasma immediately before the injection of leptin as well as 0, 10, 15, and 20 min after injection of the radioactive tracers. Immediately after collection of the final blood sample, an overdose of pentobarbital sodium (100 mg/kg) was injected through the jugular vein catheter and the animal was rapidly decapitated. The soleus, red portion of the gastrocnemius (Gastro-R), white portion of the gastrocnemius (Gastro-W), and extensor digitorum longus (EDL) muscle as well as epididymal WAT, heart muscle, and interscapular BAT were rapidly dissected and weighed. The tissue samples were homogenized at 4 °C, the homogenates were centrifuged at 4 °C, and the resulting supernatants as well as plasma samples were assayed for radioactivity. The rate constant (K_i_) of 2DG uptake in peripheral tissues was calculated using the following equation (1), as described previously^41–43^.

1 $${K}_{i}=\frac{{C}_{i}}{{\int }_{0}^{t}Cp\,dt}=\frac{{C}_{i}\cdot Kp}{C{p}_{0}\cdot (1-{e}^{-Kp\cdot t})}$$

where C_i_ is the intracellular concentration of 2- [^3^H]DG [disintegrations per min (dpm) per mg tissue (µl)] at death, Kp is the rate constant of plasma disappearance of 2-[^3^H]DG, Cp_O_ is the extrapolated plasma 2- [^3^H]DG concentration at time 0 (dpm per μl), and t is the duration of the test, i.e., 20 min. Kp is the slope at time 0 provided by a linear regression analysis. C was calculated from the radioactivities of ^3^H and ^14^C in the plasma and tissues as reported previously^42,43^.

### Immunoblot analysis

Tissue was homogenized at 4 °C in a lysis buffer containing 0.1% Nonidet P-40 and was then subjected to immunoblot analysis as described previously^30^. For analysis of the Tyr^1146^-phosphorylated form of the β subunit of IR, the homogenates were centrifuged at 4 °C and the resulting supernatants (300 µg protein) were subjected to immunoprecipitation with antibodies to IR (Cell Signalling Technology, Danvers, MA) and protein G–Sepharose (Amersham, Piscataway, NJ). The immunoprecipitates were isolated by centrifugation, washed with 0.1% Nonidet P-40 in phosphate-buffered saline, and half amounts of the immunoprecipitates were fractionated by SDS-polyacrylamide gel electrophoresis for immunoblot analysis. Separated proteins were then transferred to a polyvinylidene difluoride membrane, which was then exposed to 5% dried skim milk in a solution containing 50 mM Tris-HCl (pH 7.5), 150 mM NaCl, and 0.1% Tween 20 (TBST) before incubation for 16 h at 4 °C in TBST containing 5% bovine serum albumin and primary antibodies (1 μg/ml). For other immunoblots, tissue lysates (20 µg protein) were fractionated by SDS-polyacrylamide gel electrophoresis. Primary antibodies included those to the Tyr^1146^-phosphorylated β subunit of IR, to Ser^473^-phosphorylated Akt, to the Thr^172^-phosphorylated form of the α subunit of AMPK, to Ser^79^-phosphorylated acetyl-CoA carboxylase (ACC), and to total forms of these various proteins and α-tubulin (Cell Signalling Technology). Immune complexes were detected with horseradish peroxidase–conjugated secondary antibodies (Santa Cruz Biotechnology, Santa Cruz, CA) and enhanced chemiluminescence reagents (Amersham). The amounts of phosphorylated forms of the examined proteins were determined by densitometric scanning of immunoblots and were normalized by the corresponding amount of total protein as described previously^30^. All immunoblots for quantification are shown in Supplementary Information.

### Norepinephrine turnover

Norepinephrine (NE) turnover was measured on the basis of the decline in tissue NE content after the inhibition of catecholamine biosynthesis with α-MT as described previously^33^. α-MT (200 mg/kg) (Sigma, St. Louis, MO) was injected intraperitoneally at 6 h after leptin or saline injection into the VMH. At 0 or 2 h after α-MT injection, mice were decapitated and muscle tissue was rapidly removed and weighed. The tissue samples were then homogenized in 0.2 M perchloric acid containing 0.1 mM EDTA, and the homogenates were centrifuged at 4 °C. The NE content of the resulting supernatants was assayed by high-performance liquid chromatography (EP-300 system; Eicom, Kyoto, Japan) with a reversed-phase column (CA-5ODS, Eicom) and electrochemical detector (ECD-300, Eicom). Data are expressed as picograms of NE per milligram of tissue weight.

### Forced expression of β_2_-AR in skeletal muscle of β-less mice

The β_2_-AR was forcibly expressed in soleus and Gastro-R muscles of the right hind limb of β-less mice as described previously^30^. The mice were anesthetized with ketamine (100 mg/kg) and xylazine (10 mg/kg), and an ~1-cm incision was made in the skin around the lateral region of the gastrocnemius. The right soleus and gastrocnemius were exposed, and 20 μg of a pcDNA3.1 (Invitrogen, Carlsbad, CA) vector encoding mouse β_2_-AR under the control of the CAG promoter^44^ (provided by M. Morimatsu, Iwate University, Japan) were applied around the surface of the soleus and Gastro-R with the use of a microsyringe. Electric pulses were then administered six times at 70 V (loading period of 50 ms per pulse) from outside of the gastrocnemius with the use of an electroporator (CUY12; NEPA Gene, Ichikawa, Japan) and a pincette-type electrode (CUY650P, NEPA Gene). The left soleus and gastrocnemius muscles were subjected to the same electroporation procedure with the corresponding empty vector. The EDL of both hind limbs remained intact. The hind limb muscles were continuously cooled with ice during electroporation. Analysis of β_2_-AR expression in soleus, Gastro-R, and EDL muscles was performed 8 days after electroporation.

### RNA extraction and RT-PCR analysis

Total RNA was isolated from muscle samples with the use of Isogen (Nippon Gene, Wako, Japan). Portions of the RNA were subjected to reverse transcription (RT) with an oligo(dT) primer and avian myeloblastosis virus reverse transcriptase (Takara, Otsu, Japan), and the resulting cDNA was subjected to polymerase chain reaction (PCR) analysis with LA Taq (Takara) and primers (forward, 5′-CGAGTGGTCATCCTGATG-3′; reverse, 5′-GCAGAACTTGGAGGACC-3′) specific for β_2_-AR (Sigma Genosys, Ishikari, Japan).

### Statistical analysis

Data are presented as means ± S.E.M. and were evaluated by the unpaired or paired Student’s *t* test or by analysis of variance followed by Bonferroni’s multiple-range test. A *P* value of <0.05 was considered statistically significant.

## Electronic supplementary material

Supplementary Information
