# Supplementary material for: Induction of glucose uptake in skeletal muscle by central leptin is mediated by muscle β2-adrenergic receptor but not by AMPK
Source: Sci Rep. 2017 Nov 9;7:15141. doi: 10.1038/s41598-017-15548-6 (PMC5680211; doi:10.1038/s41598-017-15548-6)
Supplement: Supplementary file 1 — Supplementary Information [file 41598_2017_15548_MOESM1_ESM.pdf]

Supplementary Information

Induction of glucose uptake in skeletal muscle by central leptin is mediated by muscle β_2-adrenergic receptor but not by AMPK

Tetsuya Shiuchi[1,2,3], Chitoku Toda[1], Shiki Okamoto[1,2], Eulalia A. Coutinho[1,2], Kumiko Saito[1], Shinji Miura[4], Osamu Ezaki[4] & Yasuhiko Minokoshi[1,2]*

[1]Division of Endocrinology and Metabolism, Department of Homeostatic Regulation, National Institute for Physiological Sciences, National Institutes of Natural Sciences, Okazaki, Aichi 444-8585, Japan
[2]Department of Physiological Sciences, School of Life Sciences, SOKENDAI (The Graduate University for Advanced Studies), Okazaki, Aichi 444-8585, Japan
[3]Department of Integrative Physiology, Institute of Biomedical Sciences, Tokushima University Graduate School, Tokushima 770-8503, Japan
[4]Nutritional Science Program, National Institute of Health and Nutrition, Tokyo 162-8636, Japan

*Correspondence and requests for materials should be addressed to Yasuhiko Minokoshi, MD, PhD

Fig. 2a

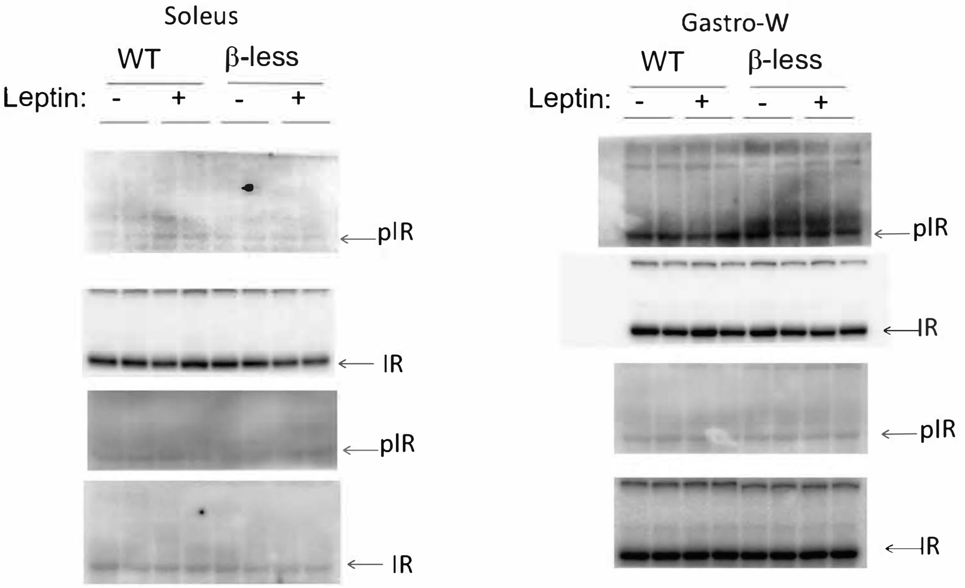

Fig. 2b

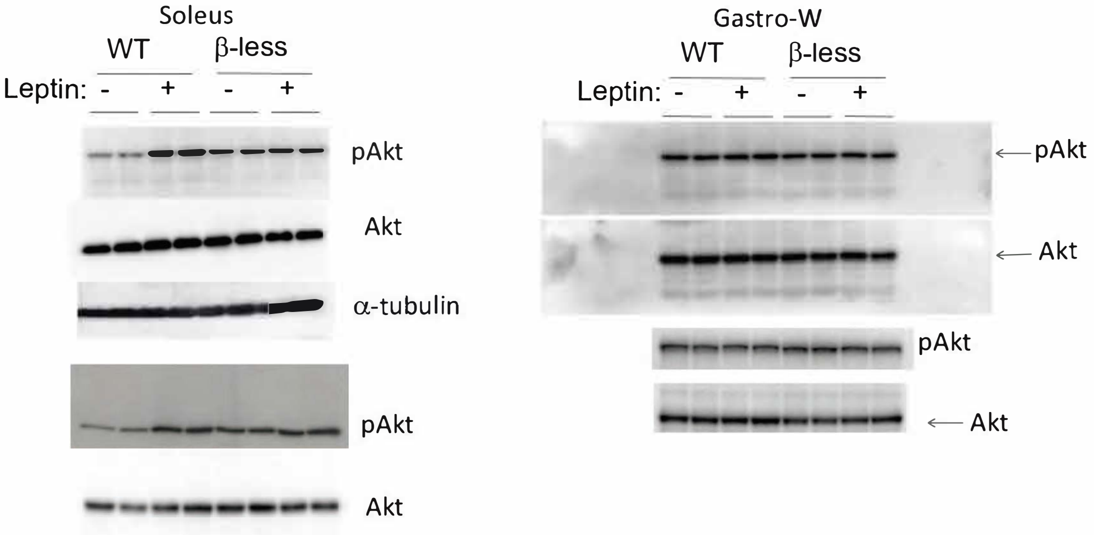

Fig. 2c

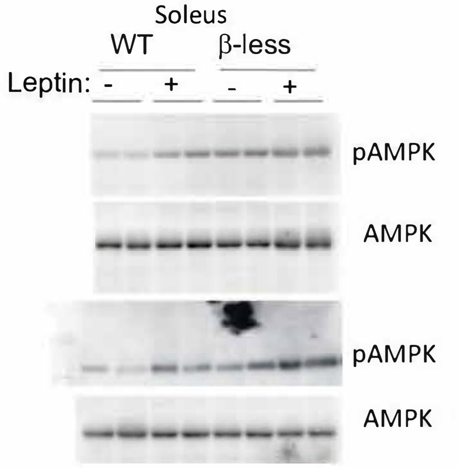

SI Figure 1. All immunoblots used for quantification in Figures 2a, 2b, and 2c.

Fig. 3c

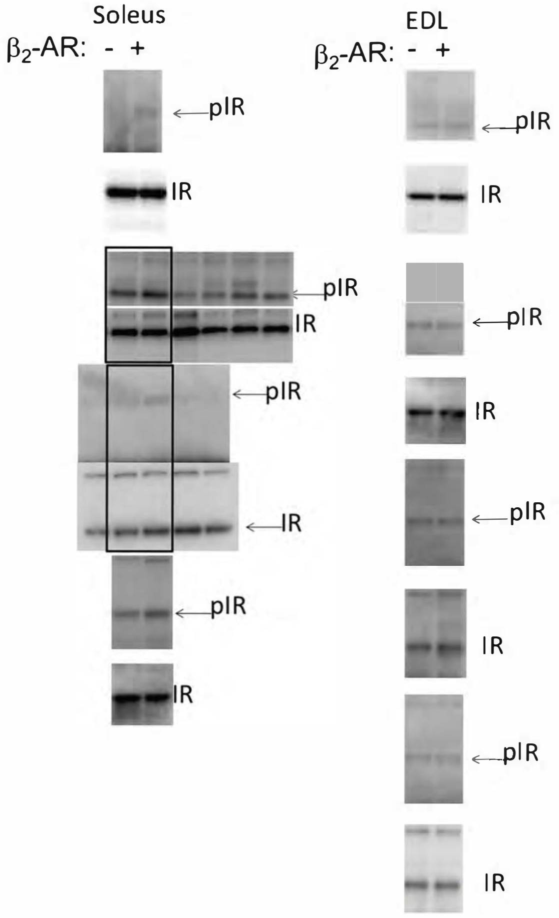

Fig. 3d

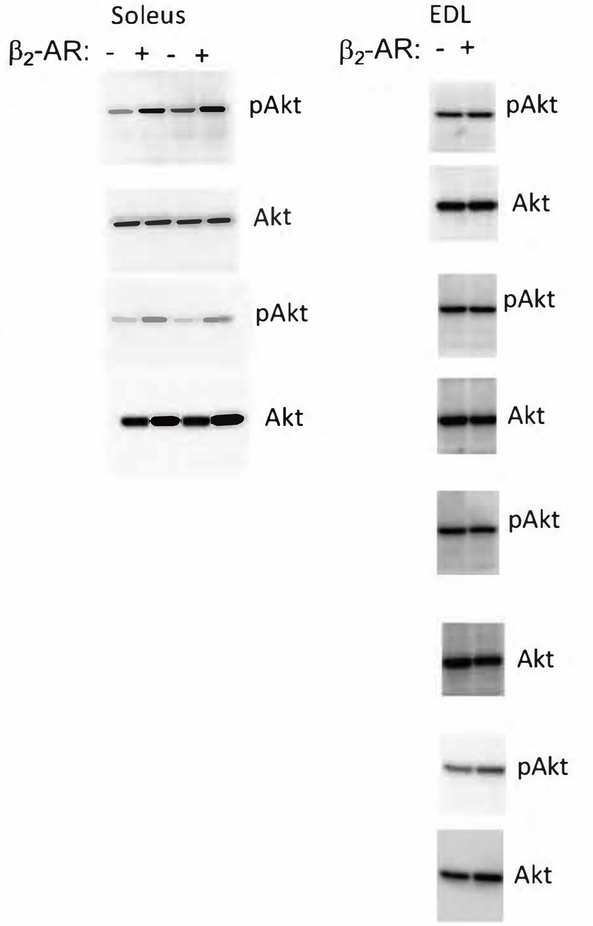

Fig. 3e

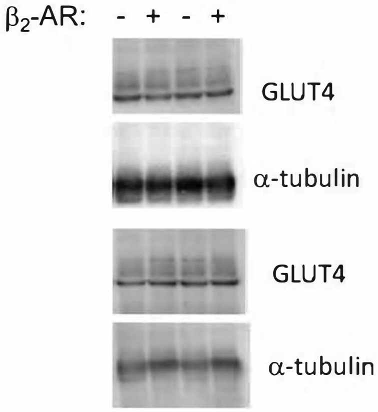

Fig. 3f

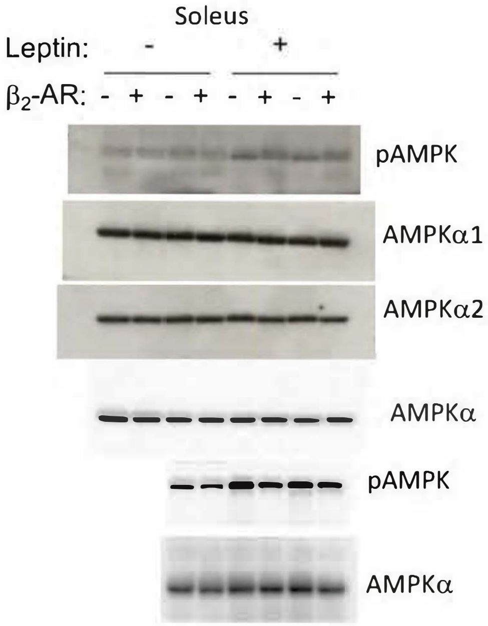

SI Figure 2. All immunoblots used for quantification in Figures 3c, 3d, 3e, and 3f.

Fig. 4a

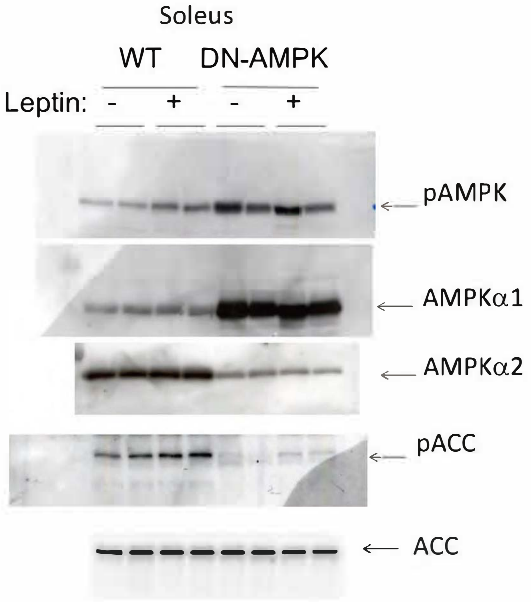

Fig. 4c

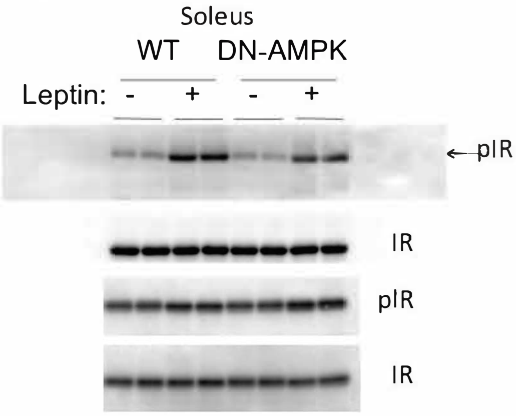

Fig. 4d

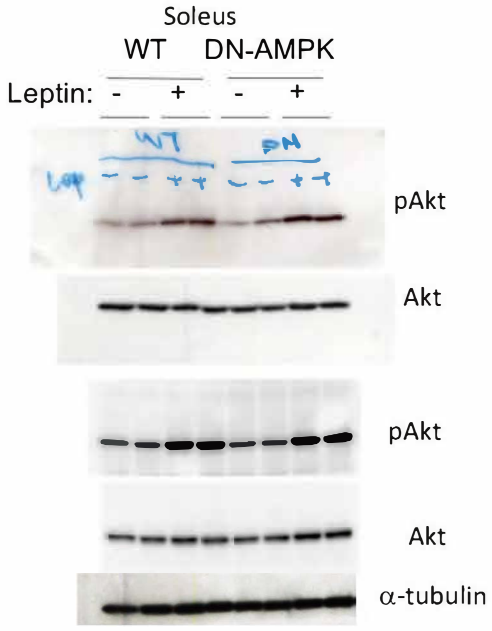

SI Figure 3. All immunoblots used for quantification in Figures 4a, 4c, and 4d.
